# Oligoprogression in Metastatic, Castrate-Resistant Prostate Cancer—Prevalence and Current Clinical Practice

**DOI:** 10.3389/fonc.2022.862995

**Published:** 2022-05-17

**Authors:** Priyanka H. Patel, Nina Tunariu, Daniel S. Levine, Johann S. de Bono, Rosalind A. Eeles, Vincent Khoo, Julia Murray, Christopher C. Parker, Angela Pathmanathan, Alison Reid, Nicholas van As, Alison C. Tree

**Affiliations:** ^1^ Department of Radiotherapy, Royal Marsden NHS Foundation Trust and Institute of Cancer Research, London, United Kingdom; ^2^ Radiology and Imaging, Royal Marsden NHS Foundation Trust, London, United Kingdom; ^3^ Drug Development Unit, Royal Marsden NHS Foundation Trust and Institute of Cancer Research, London, United Kingdom

**Keywords:** oligoprogression, stereotactic body radiotherapy, castrate resistant prostate cancer, abiraterone, enzalutamide, Androgen receptor targeted therapy, Oligoprogressive disease (OPD)

## Abstract

**Aims:**

Oligoprogression is poorly defined in current literature. Little is known about the natural history and significance of oligoprogression in patients with hormone-resistant prostate cancer on abiraterone or enzalutamide treatment [termed androgen receptor-targeted therapy (ARTT)]. The aim of this study was to determine the prevalence of oligoprogression, describe the characteristics of oligoprogression in a cohort of patients from a single center, and identify the number of patients potentially treatable with stereotactic body radiotherapy (SBRT).

**Methods:**

Castration-resistant prostate cancer (CRPC) patients who radiologically progressed while on ARTT were included. Patients with oligoprogressive disease (OPD) (≤3 lesions) on any imaging were identified in a retrospective analysis of electronic patient records. Kaplan–Meier method and log-rank test were used to calculate progression-free and overall survival.

**Results:**

A total of 102 patients with metastatic CRPC on ARTT were included. Thirty (29%) patients presented with oligoprogression (46 lesions in total); 21 (21% of total) patients had lesions suitable for SBRT. The majority of lesions were in the bone (21, 46%) or lymph nodes (15, 33%). Patients with oligoprogression while on ARTT had a significantly better prostate-specific antigen (PSA) response on commencing ARTT as compared to patients who later developed polyprogression. However, PSA doubling time immediately prior to progression did not predict OPD. Median progression-free survival to oligoprogression versus polyprogression was 16.8 vs. 11.7 months. Time to further progression after oligoprogression was 13.6 months in those treated with radiotherapy (RT) for oligoprogression vs. 5.7 months in those treated with the continuation of ARTT alone.

**Conclusions:**

In this study, nearly a third of patients on ARTT for CRPC were found to have OPD. OPD patients had a better PSA response on ART and a longer duration on ARTT before developing OPD as compared to those developing polyprogressive disease (Poly-PD). The majority of patients (70%) with OPD had lesions suitable for SBRT treatment. Prospective randomized control trials are needed to establish if there is a survival benefit of SBRT in oligoprogressive prostate cancer and to determine predictive indicators.

## 1 Introduction

Prostate cancer accounts for 26% of all new cancer cases in men, with up to 60% of cases diagnosed at a late stage in the United Kingdom (1). Castration-resistant prostate cancer (CRPC) describes a disease resistant to castration and is a life-limiting disease with a median survival of 25 months (2). Advances in treatment including the introduction of oral targeted therapies, which suppress the androgen receptor signaling pathway such as abiraterone and enzalutamide, have been proven in large, phase III randomized control trials to improve survival for CRPC patients (3–7). These androgen receptor-targeted therapies (ARTTs) give relatively minor toxicity as compared to systemic chemotherapy, maintaining quality of life and offering an additional line of treatment.

However, as with many systemic treatments, ARTT can lose efficacy over time. Patients typically develop resistant clones within 15–17 months (5, 7), with multiple mechanisms of resistance recognized (8).

Metastasis-directed therapy (MDT) for oligoprogressive disease (OPD) is a rapidly evolving potential treatment strategy. In some tumor sites, consensus guidelines have been created to characterize oligometastatic disease, but MDT for oligometastatic disease is not yet proven to improve overall survival (OS), although it has become an increasingly considered treatment option due to supporting phase II trial data (9–11). Phase III trial data are, however, awaited. OPD is still an emerging concept in prostate cancer with no prospective, randomized, phase II data on outcomes of these patients treated with or without MDT. OPD to an extent is defined by the imaging modality used, with whole-body diffusion-weighted MRI (WBDWMRI) and PET/CT being more sensitive at detecting bone progression than standard imaging (12).

OPD in broad terms includes patients with established metastatic disease with only a few lesions (usually considered as 3 or less) progressing on a background of all other metastatic sites remaining responsive to current systemic treatment (13). Patients therefore need to have shown an initial response prior to developing OPD. The hypothesis underlying treating OPD with MDT is to allow the systemic therapy to continue to work on the remaining sites, preserving the efficacy of the systemic therapy to the responsive lesions while eliminating macroscopic resistant clones with MDT. Within CRPC, this is particularly desirable when patients established on ARTT present with OPD. MDT to OPD may delay more toxic chemotherapy and improve progression-free survival (PFS) and OS. With the use of a well-tolerated treatment with a high local control rate such as stereotactic body radiotherapy (SBRT) (14–16), it is hypothesized that patient quality of life can be preserved as well. CRPC patients are often an older and frailer population in whom maintaining tolerated treatments for longer may have a significant impact on survival.

Currently, OPD is defined by the number of sites of the disease progressing. In the absence of biological hallmarks of OPD and characteristics determining prognosis, there is insufficient evidence to justify any further detailed definitions. This retrospective study aimed to quantify the prevalence of OPD in patients on ARTT and describe characteristics identified in patients with OPD on ARTT to improve the current understanding of OPD in CRPC patients.

## 2 Materials and Methods

### 2.1 Patient Population

We identified patients using a robust prescribing database from a single academic oncology center. Patients who were treated with either abiraterone or enzalutamide for metastatic CRPC from April 1, 2015, to April 30, 2017, were included. For the purpose of this study, OPD was defined as ≤3 sites of the disease progressing radiologically as compared to baseline scan after an interim biochemical, radiological, or clinical response to treatment was demonstrated. Polyprogressive disease (Poly-PD) is conversely defined as >3 sites of the disease progressing while on ARTT, with or without initial response to ARTT.

Response was defined as a prostate-specific antigen (PSA) drop of >10% from baseline PSA as per the TRAP trial protocol (NCT036446303), or a scan showing radiological response or stable disease.

Data were collected using hospital electronic patient records. All imaging for OPD patients was reviewed by an experienced consultant radiologist or nuclear medicine (NM) physician, both of whom have experience in prostate cancer imaging.

Patients were included if they had been on ARTT off trial and had radiological, biochemical, or clinical progression while on ARTT or had stopped ARTT due to other reasons such as toxicity or other medical conditions. Patients were excluded if they did not receive or had not yet progressed on ARTT. Data for those patients treated within the TRAP trial (NCT036446303), a phase II trial assessing SBRT to OPD in CRPC patients, were excluded from the time of trial entry.

### 2.2 Definition of Progression

Oligoprogression was radiologically defined using a combination of adapted Prostate Cancer Working Group 3 (PCWG3) (17), Response Evaluation Criteria in Solid Tumors (RECIST) v 1.1 (18), and Metastasis Reporting and Data System for Prostate Cancer (METRADS-P) (19). This reflects the range of imaging performed in a “real world” scenario.

For CT, it is the development of any new lesion or ≥20% increase in diameter of an existing lesion compared to the nadir or baseline CT scan; for bone scan, 2 or more new bone lesions or worsening of an existing lesion with a rising PSA on NM bone scan; for MRI, new or recurrent lesion compared to the nadir, unequivocal increase in size compared to a baseline scan, or regions with high signal intensity on high b value images on WBDWMRI; and for PET, any new avid lesion with standardized uptake value (SUV) above background or >20% increase in SUV max, compared to baseline/nadir scan on PET/CT, as used in clinical practice (18–20).

Further progression beyond OPD was defined as any further growth of OPD lesions or other lesions on CT, increase in SUV on PET/CT, or changes in signal intensity on WBDWMRI scans suggesting further progression and/or appearance of any new lesions compared to nadir/baseline scan, associated with PSA progression.

PSA progression was defined as a PSA increase of 25% from the nadir plus an absolute increase of 2 ng/ml, as per PCWG guidelines (17).

### 2.3 Definition of Endpoints

PFS 1 (PFS1) was defined as the time from starting ARTT to radiological progression or censored to the last follow-up.

PFS 2 (PFS2) was defined as the time between OPD detection and further radiological progression or death or censored to the last follow-up.

SBRT suitability of OPD lesions was determined by proximity to critical structures, size of lesion <6 cm, and index lesion not previously being irradiated.

OS was defined as the time from progression on ARTT to death of any cause or censored to the last follow-up.

PSA doubling time was calculated using the nadir PSA after starting ARTT and PSA at diagnosis of OPD.

Data were analyzed using the Kaplan–Meier survival curves and log-rank tests, and univariate and multivariate logistic regression using GraphPad version 9.0.

## 3 Results

### 3.1 All Patients on Androgen Receptor-Targeted Therapy

A total of 102 patients met the inclusion/exclusion criteria. All patients initiated ARTT off trial for CRPC. The median follow-up from starting ARTT was 35.7 months.

Baseline characteristics for patients at initial diagnosis and at starting ARTT are summarized in **Tables 1**, **2**. Characteristics at the time of progression, stratified by OPD vs. Poly-PD criteria, are shown in **Table 3**.

**Table 1:** Baseline characteristics at initial diagnosis.

Characteristics at initial diagnosis	N = 102
	
Age (years), n (%)	
Median (IQR)	67 (61–73)
≤60	22 (22)
61–70	44 (43)
>70	36 (35)
NCCN stage, n (%)	
Very low	1 (1)
Low	0
Intermediate	15 (15)
High	32 (32)
Very high	20 (20)
Metastatic	34 (33)
PSA (ng/ml), n (%)	
Mean ( ± SD)	227 ± 812
<10	23 (22)
10–20	22 (22)
>20	57 (56)
Primary radical treatment, n (%)	
Radical prostatectomy	11 (11)
Radical radiotherapy plus ADT	60 (59)
Postoperative radiotherapy	2 (2)
HIFU	1 (1)

ARTT, androgen receptor-targeted therapy; PSA, prostate-specific antigen; IQR, interquartile range; NCCN, National Comprehensive Cancer Network; ADT, androgen deprivation therapy; HIFU, high-intensity focus ultrasound.

**Table 2:** Characteristics at starting ARTT.

Characteristics at starting ARTT	N = 102
Age years, n (%)	
Median (IQR), years	77 (71–82)
≤60 years	3 (3)
61–70 years	17 (17)
>70 years	82 (80)
Mean PSA, ng/ml ( ± SD)	171 ± 602
Prior docetaxel, n (%)	
Yes	8 (8)
No	94 (92)
Line of metastatic therapy, n (%)*	
2nd line	37 (36)
3rd line	32 (32)
4th line	33 (32)
ARTT treatment, n (%)	
Abiraterone	62 (60)
Enzalutamide	40 (40)

ARTT, androgen receptor-targeted therapy; PSA, prostate-specific antigen; IQR, interquartile range.

^*^Lines of therapy include treatments such as luteinizing hormone-releasing hormone agonists or antagonists (LHRH), combined androgen blockade, dexamethasone, and docetaxel chemotherapy.

**Table 3:** Characteristics at progression on ARTT; OPD vs. Poly-PD.

Characteristics at progression	OPD, N = 30	Poly-PD, N = 52
Age (years), n (%)		
Median (IQR), years	75 (70–83)	79 (72–83)
≤60	1 (3)	1 (2)
61–70	7 (23)	8 (15)
>70	22 (73)	43 (83)
PSA mean, ng/ml ( ± SD)		
At prostate cancer diagnosis	120 ± 236	360 ± 1,100
At starting ARTT	63 ± 129	191 ± 751
At progression	15 ± 14	168 ± 434
Prior docetaxel, n (%)		
Yes	2 (7)	2 (4)
No	28 (93)	50 (96)
Line of therapy, n (%)		
2nd line	12 (40)	19 (37)
3rd line	12 (40)	14 (26)
4th line	6 (20)	19 (37)
ARTT treatment, n (%)		
Abiraterone	21 (70)	33 (63)
Enzalutamide	9 (30)	19 (37)

ARTT, androgen receptor-targeted therapy; PSA, prostate-specific antigen; IQR, interquartile range; OPD, oligoprogressive disease; WBDWMRI, whole-body diffusion-weighted MRI.

### 3.2 Oligoprogressive Disease Patients

**Figure 1:**
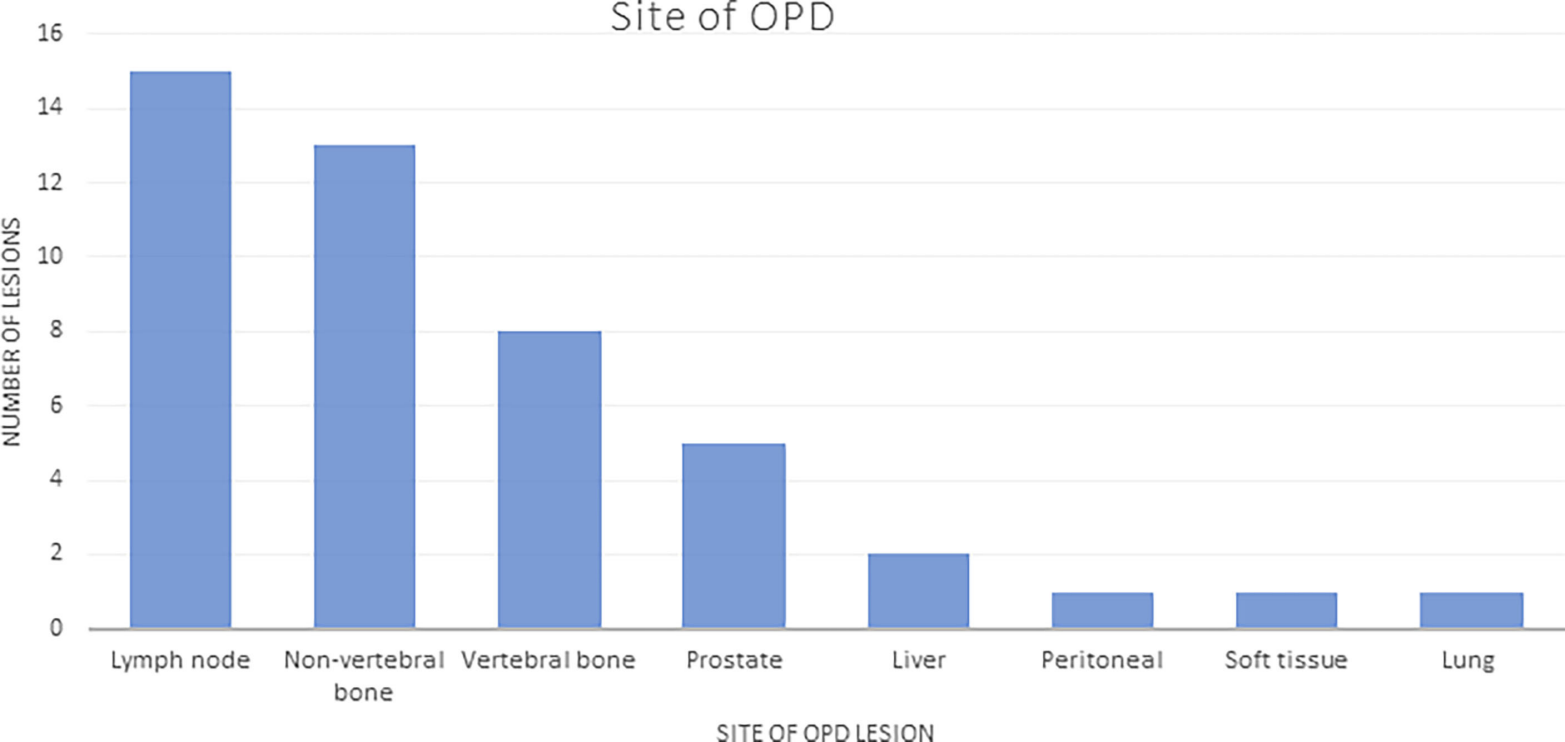
Sites of OPD: number of lesions listed by site (total number of lesions in patients with OPD = 46). OPD, oligoprogressive disease.

Of the 102 patients, 82 (80%) had radiological evidence of progression, and 30 (29%) patients progressed with ≤3 sites (OPD) with 46 lesions in total, based on WBDWMRI, choline PET/CT, CT, or bone scan (**Table 4**). The most common sites of OPD were in bone lesions (20). Five patients had prostate oligoprogression (**Figure 1**).

**Table 4:** Characteristics of OPD patients.

Characteristics of OPD patients	OPD N = 30
NCCN stage at diagnosis, n (%)	
Intermediate	4 (13)
High	7 (23)
Very high	11 (37)
Metastatic	8 (27)
Primary radical treatment, n (%)	
Radical prostatectomy	5 (17)
Radical radiotherapy plus ADT	16 (53)
Postoperative radiotherapy	4 (13)
Salvage prostatectomy	2 (7)
Synchronous metastases, n (%)	8 (27)
Metachronous metastases	22 (73)
Oligometastatic (at initial metastatic diagnosis)	20 (67)
Polymetastatic	10 (33)
Oligometastatic at starting ARTT, n (%)	12 (40)
Polymetastatic at starting ARTT, n (%)	18 (60)
Number of OPD lesions, n (%)	
1 lesion	17 (57)
2 lesions	10 (33)
3 lesions	3 (10)
Scan detecting OPD, n (%)	
WBDWMRI	6 (20)
Choline PET/CT	6 (20)
CT	16 (53)
Bone scan	2 (7)
1st follow-up scan after diagnosis of OPD, n (%)	
WBDWMRI	5 (17)
Choline PET/CT	4 (13)
CT	8 (27)
Bone scan	1 (3)
CT and bone scan	4 (13)
TRAP trial imaging	3 (10
MRI pelvis	2 (7)
No imaging	3 (0)

ADT, androgen deprivation therapy; ARTT, androgen receptor-targeted therapy; NCCN, National Comprehensive Cancer Network; OPD, oligoprogressive disease; PSA, prostate-specific antigen; IQR, interquartile range; WBDWMRI, whole-body diffusion-weighted MRI.

The majority of the OPD lesions (36/46, 78%) were suitable for SBRT; 21/30 (70%) patients had all OPD sites suitable for SBRT. The reasons for lesions not being suitable for SBRT included the following: previously irradiated lesions (n = 4, 9%); lesions too large for SBRT in the liver, ischial bone metastasis, and para-spinal soft tissue mass (n = 3, 6%); ill-defined lesions in 2 patients with disease in Gerota’s fascia and disease encasing the right ureter (n = 2, 4%); and a single lesion that required urgent radiotherapy (RT) to spinal disease causing nerve root compression (n = 1, 2%). Two-thirds of patients (20, 70%) with OPD had oligometastatic disease (defined as ≤5 lesions) at initial diagnosis. At the time of starting ARTT, the prevalence of oligometastatic disease had reduced to 12 (40%) patients.

#### 3.2.1 Progression-Free Survival 2 (From Oligoprogressive Disease to Further Progression/Death)

Twenty-seven of 30 (90%) OPD patients were followed up with imaging bone scan and CT scan, WBDWMRI, or PET/CT (**Table 4**). Three patients had no follow-up imaging due to no PSA progression after switching from prednisolone to dexamethasone (1), death (1), and treatment elsewhere (1). The median time to the next follow-up scan after OPD is 13.1 months.

**Figure 2:**
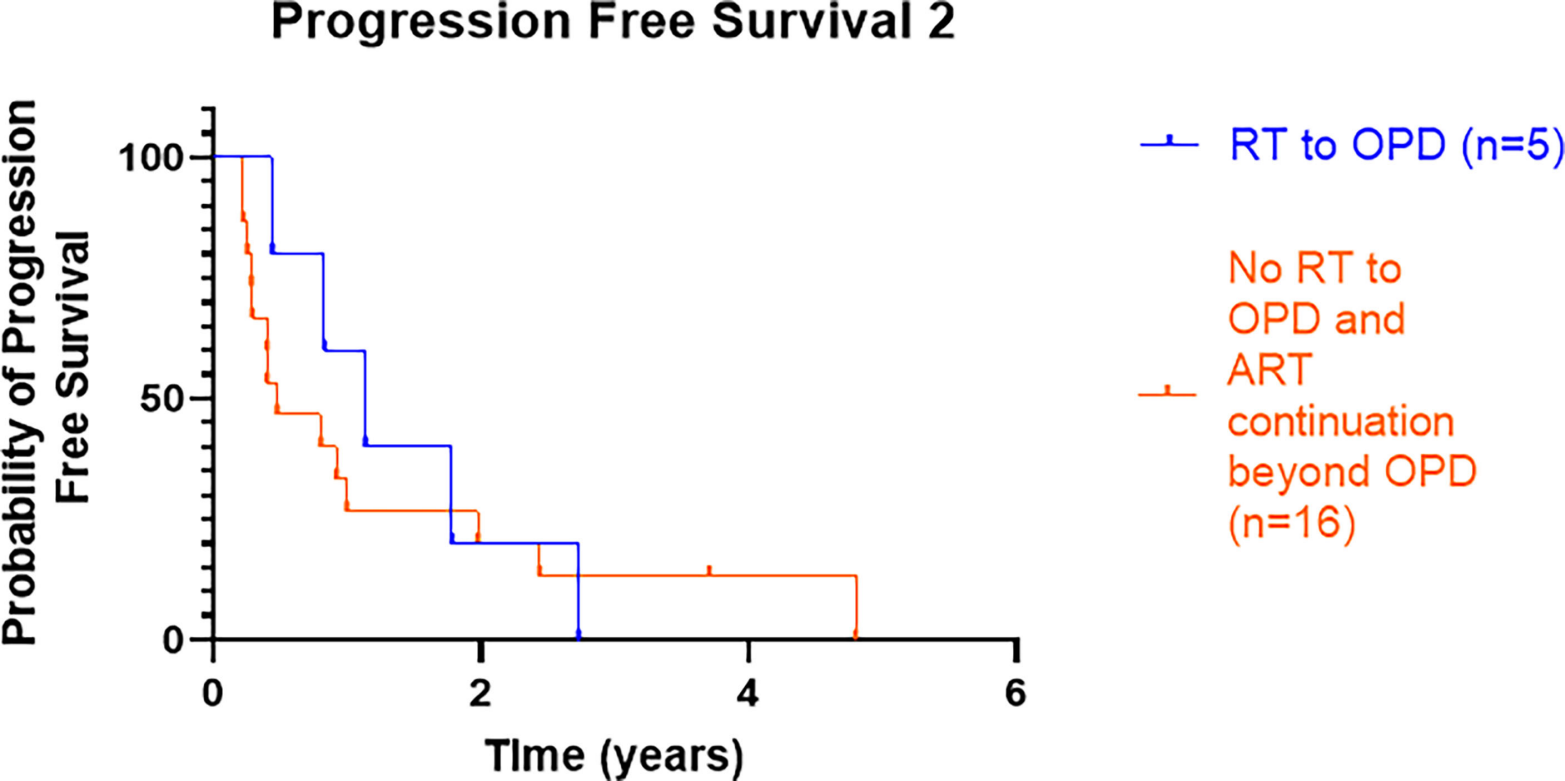
Kaplan–Meier curve presenting PFS2 from OPD to further radiological progression or death for those patients treated with RT with or without continuation of ARTT or continuation of ARTT alone. PFS2, progression-free survival 2; OPD, oligoprogressive disease; RT, radiotherapy; ARTT, androgen receptor-targeted therapy.

A small proportion of patients (8/30, 27%) had RT to OPD sites; 3 (10%) were treated within the TRAP trial, and data from trial entry have been excluded from the results. Three of the 5 patients treated off trial received SBRT (30 Gy in 3–5 fractions) to lymph nodes and bone metastasis, and 2 patients received palliative RT: one patient received 24 Gy in 4 fractions to the prostate, and the other patient received 20 Gy in 5 to the sacrum. The median PFS2 in those patients treated with RT was 13.6 months (n = 5), and in patients who did not receive RT for OPD but continued ARTT alone, it was 5.7 months (n = 16). There was no overt difference between the two groups, with log-rank HR 0.8 (95% CI 0.3–2.1), p = 0.68 (**Figure 2**).

Of the 5 patients who received RT to OPD, PFS2 was shorter for the 2 patients who received palliative RT doses compared to the 3 patients who received radical SBRT doses (7.6 vs. 21.4 months, respectively), p = 0.039. One patient who received 24 Gy in 4 fractions discontinued enzalutamide after commencing RT. Four out of the 5 patients had a high burden of disease defined as >3 known sites of the disease since initial prostate cancer diagnosis (i.e., not oligometastatic disease); however, only OPD sites were irradiated.

Of the five patients who had RT to OPD, one patient treated for prostate OPD had no further imaging performed; this patient had oligometastatic disease. Two patients eventually progressed in ≤3 sites (repeat OPD): one patient had re-occurrence in the irradiated sacrum (treated with 20 Gy in 5 fractions), and the other patient who received SBRT to a para-aortic lymph node received repeat SBRT to two more oligoprogressing lymph nodes. Two patients who received RT to OPD lesions in the prostate and the lymph nodes (para-aortic and common iliac) progressed with Poly-PD subsequently.

Of the 16 patients who did not receive RT but continued ARTT, 8 (50%) patients had further OPD (i.e., ≤3 lesions including original OPD lesion progressing) on subsequent radiological imaging, and one of these patients subsequently was treated within the TRAP trial. Five (31%) patients had Poly-PD, and 3 (19%) patients had not radiologically progressed.

Six (28%) patients not treated with RT did not continue ARTT beyond OPD.

### 3.3 Oligoprogressive Disease Versus Polyprogressive Disease

Fifty-two (51%) patients had radiological evidence of Poly-PD with >3 sites of progression. The remaining 20 patients stopped ARTT due to reasons including PSA progression alone without imaging, toxicity, or death due to other causes. Five patients (10%) with Poly-PD continued ARTT beyond progression.

#### 3.3.1 Progression-Free Survival 1 (Starting Androgen Receptor-Targeted Therapy to Radiological Progression)

**Figure 3:**
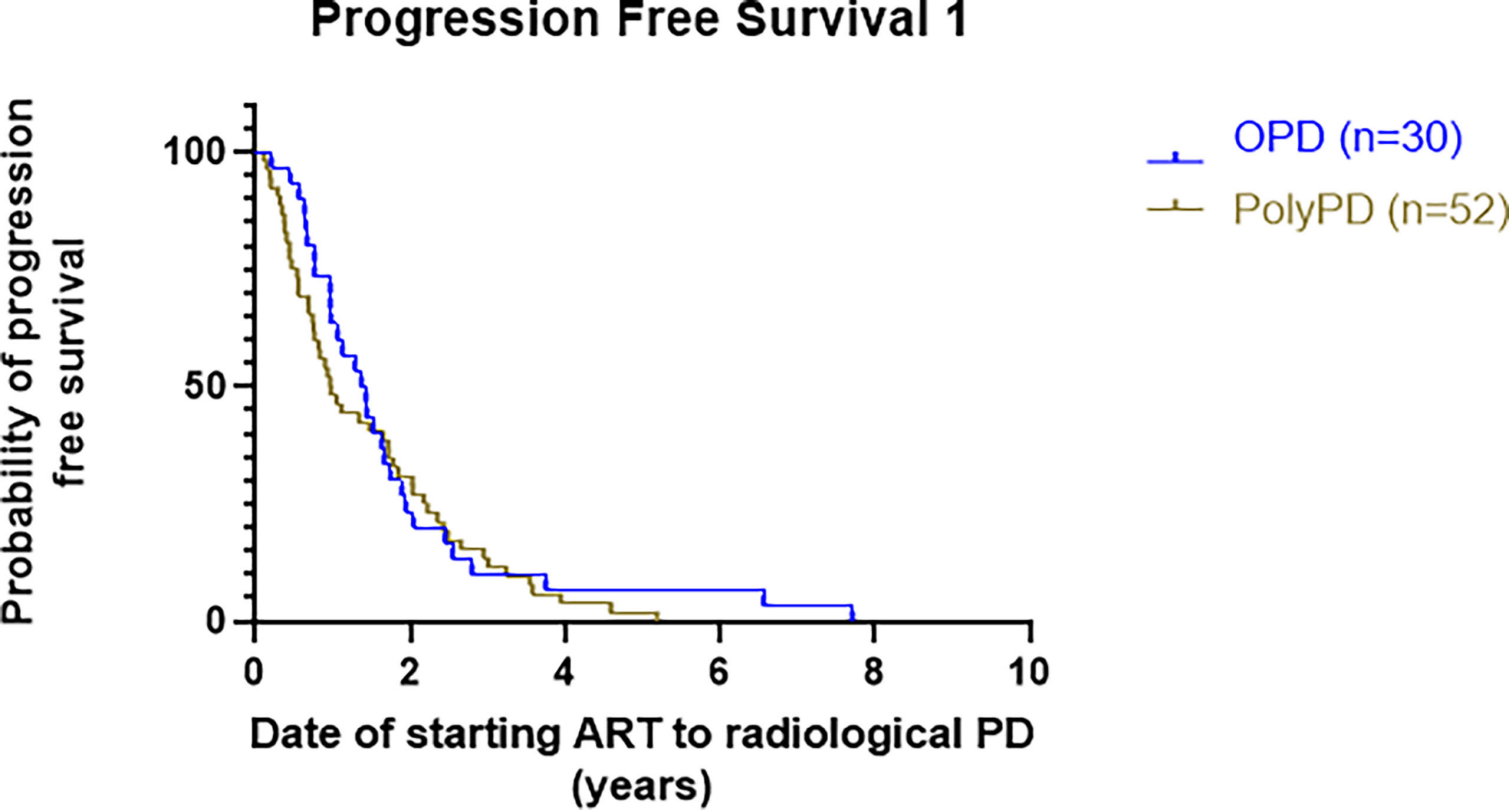
Kaplan–Meier curve presenting PFS1 of patients with OPD versus Poly-PD. PFS1 is calculated as time from starting ARTT to either OPD or Poly-PD. PFS1, progression-free survival 1; OPD, oligoprogressive disease; Poly-PD, polyprogressive disease; ARTT, androgen receptor-targeted therapy.

Median PFS1 was 16.8 months in patients with OPD, while the median time to Poly-PD from starting ARTT was 11.7 months, with no overt difference, log-rank HR 0.84 (95% CI 0.53–1.3), p = 0.43 (**Figure 3**).

#### 3.3.2 Overall Survival: Oligoprogressive Disease Versus Polyprogressive Disease

Median OS was similar in patients with prostate, lymph node, or bone OPD: prostate (23 months), lymph node (24.7 months), and bone (24 months) but better than visceral OPD (16.5 months), with no overt difference between each site (p = 0.19). One patient presented with OPD within the liver but progressed rapidly within 6 weeks with widespread progression.

**Figure 4:**
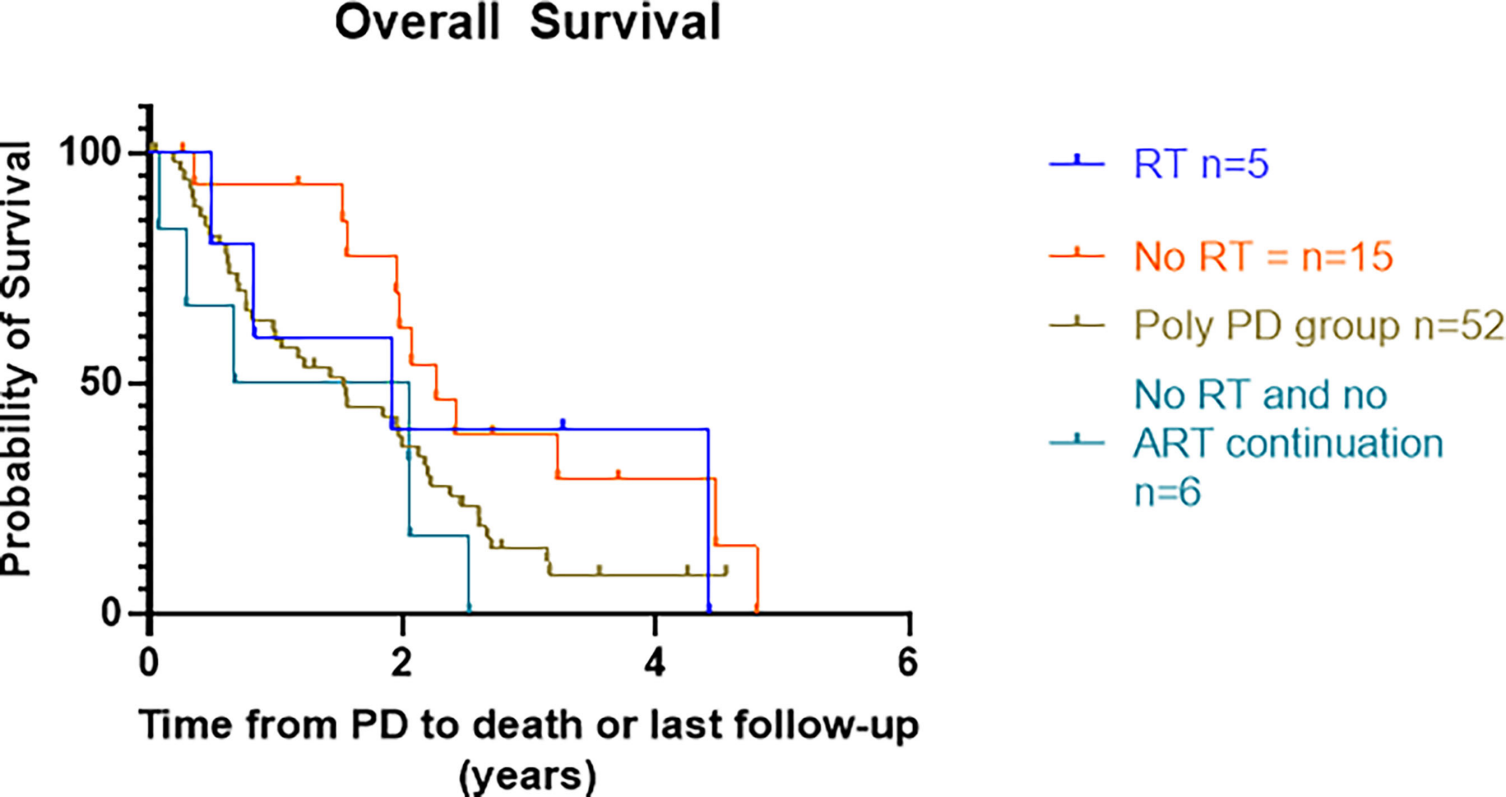
Kaplan–Meier curves presenting OS from date of progression on ARTT to death or last treatment groups. OS, overall survival; ARTT, androgen receptor-targeted therapy.

Median OS in the RT group was 22.9 months (53 months in the SBRT only group). In patients receiving no RT, the median OS for those who continued ARTT vs. no continuation of ARTT was 27.2 vs. 16.3 months, p = 0.03 (**Figure 4**).

### 3.4 Prostate-Specific Antigen Kinetics: Oligoprogressive Disease Versus Polyprogressive Disease

The median [interquartile range (IQR)] PSA doubling time prior to progression was similar in both the OPD group at 5.5 (3.9–8.8) months and the Poly-PD group at 5.2 (2.9–9.3) months. The PSA response to ARTT in the OPD group was better than in the Poly-PD group with a median (IQR) percentage reduction of 89% (67%–95%) in the OPD group as compared to 70% (40%–84%) in the Poly-PD group. Of the 5 patients who received RT off trial for OPD, one patient who received SBRT did not have a PSA response but remained radiologically stable on ARTT for 21 months.

Both univariate and multivariate logistic regression analyses indicated an association between OPD and percentage PSA decline at the nadir with an odds ratio (OR) of 0.028 (CI 0.002–0.20), p < 0.0001. There was no overt association found with age, OPD, PSA doubling time, line of therapy, National Comprehensive Cancer Network (NCCN) stage at diagnosis, and type of ARTT.

### 3.5 Summary

In this study, 86 patients who progressed on ARTT had imaging available at the time of progression. Thirty (35%) of these patients presented with OPD during their ARTT treatment course. A substantial proportion of patients on ARTT (23%) had OPD lesions suitable for treatment with SBRT and would have potentially been eligible for entry into the TRAP trial, a prospective phase II single-arm study.

## 4 Discussion

MDT for OPD is an evolving treatment paradigm with no prospective or retrospective evidence as compared with the standard of care in prostate cancer patients. Despite this, some patients receive MDT for OPD in this clinical setting, although within the United Kingdom, SBRT for OPD is not yet commissioned.

Median PFS2 was >11 months longer in the SBRT plus ARTT patients as compared to ARTT alone for OPD, with a median PFS2 of 17.2 months. The numbers in this cohort, however, are very small; therefore, it is difficult to draw any conclusions. However, this reflects real-world practice where patients are being treated with RT for OPD, highlighting the need for a randomized control trial. Furthermore, a substantial number of OPD patients continued ARTT beyond OPD compared to those who continued beyond Poly-PD. This practice is in keeping with PCWG3 guidance (17) to continue ARTT until no further symptomatic benefit or until significant radiological progression. The difference in median OS between patients who continued ARTT beyond OPD (without MDT) and Poly-PD was >5 months, suggesting that ARTT alone beyond OPD may improve patient outcomes, and therefore, a trial comparing continuation of ARTT with vs. without SBRT for OPD is crucial in determining the magnitude of effect.

Data from retrospective studies to date have reported minimal toxicity, with 2 studies reporting 2 patients with grade 3 toxicity and no studies reporting ≥grade 4 toxicity (21–25). Deek et al. (26) in a retrospective series including 68 patients treated with MDT for OPD reported the time to next intervention as 15.6 months (systemic or further RT) and a median distant metastasis-free survival of 10.8 months. Fifty-five (80.9%) patients stayed on the same systemic therapy at the time of MDT. However, all patients included had MDT to OPD ± non-OPD sites. Fifty (74%) patients had ≤3 metastatic sites (i.e., oligometastatic disease) at baseline. A multicenter retrospective study by Detti et al. (22) included 32 patients on abiraterone, not suitable for chemotherapy, treated with RT to OPD lesions or for palliative intent to treat symptoms, and median PFS2 was 9.6 months. A retrospective multicenter study by Triggiani et al. (27) included 86 patients with bone or lymph node OPD lesions (up to 5) treated with SBRT for patients on 1st-line treatment with ADT. The study found a median new metastasis-free survival of 12.3 months, with 26 of the patients undergoing further SBRT. The studies all suggest a prolonged progression-free interval after SBRT to sites of OPD. However, there is marked heterogeneity among these trials with regard to inclusion criteria and RT administration, highlighting the lack of consensus on defining and therefore treating OPD patients. There is also no comparison to the standard of care to determine the magnitude of benefit. This is needed to ensure that the apparently encouraging PFS2 intervals are not solely due to optimal case selection.

A difference between median OS in the SBRT group (53 months) and those with OPD and no continuation of ARTT (16.3 months) within this study could also be due to selection bias. Patients clinically deteriorating would not have been suitable for SBRT and therefore favored to switch to a different systemic therapy or best supportive care, with asymptomatic patients with a better performance status more likely to be treated with SBRT.

Twelve patients found to have OPD within this study were identified on functional imaging such as PET/CT or WBDWMRI instead of standard imaging using CT or bone scans. These scanning methods are not the standard of care in the United Kingdom; however, they are considered useful in the early detection of OPD in clinical practice. Yoshida et al. (23) studied 23 patients with CRPC who underwent WBDWMRI scans identifying OPD, which were then treated with RT doses ranging from 60 to 78 Gy (2 Gy per fraction) to the prostate/lymph node metastasis and 30 to 39 Gy (2–3 Gy per fraction) to bone metastases. The study reported the median time to PSA failure to be 8.7 months. However, there is no comparison with standard CT and bone scan imaging. Detection of progression on bone scan is notoriously difficult, hence the interest in next-generation imaging such as WBDWMRI and PET/CT. Although these imaging modalities are impacting patient treatment decisions, the significance of early detection on these scans is not known. Defining the eligibility imaging required for a randomized trial will likely set the standard going forward.

Patients with OPD had a significantly lower PSA at the nadir on ARTT, with a median reduction in PSA > 20% than in patients with Poly-PD. Median time on ARTT before progressing was also 5 months longer in patients presenting with OPD compared to Poly-PD. These data suggest that OPD may be more prevalent in those patients who have had a significant and sustained PSA response to ARTT. Close imaging surveillance of these patients may help to identify OPD, facilitating MDT before widespread metastatic disease develops. Surprisingly, PSA doubling time at progression did not predict OPD. A lower median PSA at the nadir in the OPD group of 2.6 vs. 9 in the Poly-PD group may have obscured the effect of PSA doubling time, with the OPD group requiring a smaller overall rise to double as compared to the Poly-PD group. Larger datasets are required before PSA response criteria identifying likely OPD can be proposed.

PSA change in response to RT within this study was not a useful biomarker predicting response to MDT. Circulating tumor DNA (ctDNA) may be a more discriminatory marker in predicting response to SBRT, in combination with PSA results, and is a necessary component of any prospective trial to ensure appropriate patients are selected for treatment (28).

We acknowledge that there are a number of limitations, with this study being retrospective. The study includes a small number of heterogeneous patients treated with RT; therefore, this limits the strength of the conclusions drawn. Treatment paradigms were not protocolized; hence, the imaging and RT delivered are varied, and outcomes may reflect selection bias rather than underlying biology. The study reflects real-world practice and highlights characteristics within a cohort of patients who may be eligible for treatment with SBRT and helps to delineate the population for further study.

Ongoing clinical trials include the TRAP trial (NCT036446303) treating CRPC patients on ARTT with SBRT to up to 2 OPD lesions, with a biomarker assessment panel evaluating the use of WBDWMRI and ctDNA in predicting response. MEDCARE (NCT 04222634) is a phase II study assessing the role of prostate-specific membrane antigen (PSMA) PET-CT in OPD, which may tell us which patients would benefit from SBRT to OPD in CRPC (**Table 5**). With patients now accessing ARTT in the hormone-sensitive setting (29), the question of whether RT can improve progression-free and OS needs to include patients progressing on first-line therapy, to ensure the relevance of conclusions for future patients.

**Table 5:** Summary of clinical trials in progress utilizing metastasis-directed therapy for oligoprogression metastatic prostate cancer.

Clinical trial	Phase	N	OPD definition	Treatment arms	Systemic therapy at the time of OPD	Primary outcome	Estimated study completion date	Clinical trials.gov identifier
TRAP	II	84	1–2 OPD lesions in bone, lymph node, prostate, or lung	SBRT to OPD lesions and continuation of ARTT	Enzalutamide/abiraterone	PFS	February 2022	NCT036446303
IOSCAR	Pilot	40	1–3 new or OPD lesions or oligopersistent lesions in bone or lymph node	SBRT to oligorecurrent alone or OPD + ADT	ADT for OPD No ADT for oligorecurrent disease	Immune response evaluation	October 2024	NCT04624828
MEDCARE	II	18	≤3 extracranial metastases or local recurrence	SBRT or metastatectomy to OPD lesion and continuation of systemic therapy	ADT alone or in combination with another systemic therapy	Next-line systemic treatment-free survival and PSMA PET-CT accuracy and predictive value	January 2030	NCT04222634
NCT04838899	I	30	≤5 OPD lesions or PSA progression only in the setting of oligometastases (≤5 metastatic lesions). With ≤3 metastases in one organ	SBRT + abiraterone	Abiraterone	SBRT-related toxicity and PFS	December 2025	NCT04838899

ARTT, androgen receptor-targeted therapy; OPD, oligoprogressive disease; SBRT, stereotactic body radiotherapy; ADT, androgen deprivation therapy; PFS, progression-free survival; PSMA, prostate-specific membrane antigen; OPD, oligoprogressive disease.

Oligoprogression is common in a real-world setting. We identified a subgroup of patients potentially suitable for a novel treatment strategy including SBRT. Ongoing trials will help identify predictive biomarkers, but a randomized trial is needed to establish if there is a clinically significant benefit after SBRT.

## Data Availability Statement

The datasets presented in this article are not readily available because data sharing will need to be requested through the official hospital channels. Requests to access the datasets should be directed to PP.

## Ethics Statement

Ethical review and approval were not required for the study on human participants in accordance with the local legislation and institutional requirements. Written informed consent for participation was not required for this study in accordance with the national legislation and the institutional requirements.

## Author Contributions

PP project development, data collection, data analysis, and manuscript writing. NT and DL: radiology review, data collection, and manuscript writing. CP, JM, AP, AR, NvA, RE, VK, and JdB manuscript writing. AT: project development and manuscript writing. All authors listed have made a substantial, direct, and intellectual contribution to the work and approved it for publication.

## Funding

This paper represents independent research part-funded by the National Institute for Health Research (NIHR) Biomedical Research Centre at the Royal Marsden NHS Foundation Trust and the Institute of Cancer Research. AT acknowledges support from Prostate Cancer UK, Cancer Research UK (C33589/A28284 and C7224/A28724 CRUK RadNet), and the JP Moulton Foundation. All authors: this project represents independent research supported by the National Institute for Health Research (NIHR) Biomedical Research Centre at The Royal Marsden NHS Foundation Trust and the Institute of Cancer Research, London.

## Author Disclaimer:

The views expressed are those of the author(s) and not necessarily those of the NHS, the NIHR, or the Department of Health.

## Conflict of Interest

AT has received research funding and/or travel funding from Elekta, Varian, and Accuray, and honoraria/travel budget support from Janssen and historically from Astellas. AP has received research funding from Elekta and honoraria from Elekta and Janssen. JB has served on advisory boards and received fees from many companies including Amgen, Astra Zeneca, Astellas, Bayer, Bioxcel Therapeutics, Boehringer Ingelheim, Cellcentric, Daiichi, Eisai, Genentech/Roche, Genmab, GSK, Harpoon, ImCheck Therapeutics, Janssen, Merck Serono, Merck Sharp & Dohme, Menarini/Silicon Biosystems, Orion, Pfizer, Qiagen, Sanofi Aventis, Sierra Oncology, Taiho, Terumo, and Vertex Pharmaceuticals. He is an employee of ICR and has received funding or other support for his research work from AZ, Astellas, Bayer, Cellcentric, Daiichi, Genentech, Genmab, GSK, Janssen, Merck Serono, MSD, Menarini/Silicon Biosystems, Orion, Sanofi Aventis, Sierra Oncology, Taiho, Pfizer, and Vertex, which has a commercial interest in abiraterone, PARP inhibition in DNA repair defective cancers, and PI3K/AKT pathway inhibitors (no personal income). JB was named as an inventor, with no financial interest for patent 8,822,438, submitted by Janssen, that covers the use of abiraterone acetate with corticosteroids. He has been the CI/PI of many industry-sponsored clinical trials. JB is a National Institute for Health Research (NIHR) Senior Investigator. CP served on Bayer in the education steering committee on January 21, 2020. He received payment from ICR for the podcast on mCRPC, July 2021; speaker fees from Janssen for Summit on Feb 2020, lecture on Nov 2020, and Ad board on March 2020; fees from Clarity Pharmaceuticals for Ad board on April 2020; fees from Myovant for Ad board on October 2020; fees from ITM Radiopharma for Ad board on October 2021; payment from AAA to ICR for Ad board on Dec 2021. RE declares receiving honorarium as a speaker at GU-ASCO Meeting 2016 and support from Janssen, as a speaker at the Royal Marsden NHS Foundation Trust 2017. She has received an honorarium as a speaker at the University of Chicago in 2018 and Bayer and Ipsen honoraria at the ESMO meeting in 2019, and she is a member of the external expert committee of AstraZeneca UK Limited. PP received an honorarium for educational cases for PinPoint Case Platform, Mirrors of Medicine, and research post funded by the Royal Marsden/Institute of Cancer Research Biomedical Research Centre and Prostate Cancer UK. N.J.V.A. declares consultant honorarium from Accuray and Research funds from Accuray. AR has received honoraria and travel support from Janssen, Astellas and AZD. VK reports honoraria for speakers bureaus, personal fees and non-financial support from Accuray, Astellas, Bayer, Boston Scientific, and Janssen.

The remaining authors declare that the research was conducted in the absence of any commercial or financial relationships that could be construed as a potential conflict of interest.

## Publisher’s Note

All claims expressed in this article are solely those of the authors and do not necessarily represent those of their affiliated organizations, or those of the publisher, the editors and the reviewers. Any product that may be evaluated in this article, or claim that may be made by its manufacturer, is not guaranteed or endorsed by the publisher.
